# Dataset on India’s imports of *Penaeus vannamei* brooders from the United States

**DOI:** 10.1016/j.dib.2025.112185

**Published:** 2025-10-18

**Authors:** M.C. Remany, Daly Cyriac, V. Shanmuga Arasu, Anup Mandal

**Affiliations:** a Aquatic Quarantine Facility Rajiv Gandhi Centre for Aquaculture, MPEDA, (Ministry of Commerce and Industry, Government of India), TNFDC Hatchery Complex, Kapaleeswarar Nagar, Neelankarai, Chennai 600 115, Tamil Nadu, India; b Rajiv Gandhi Centre for Aquaculture, MPEDA, 3/197, Poompuhar Road, Karaimedu Village, Sirkazhi Taluk- 609 109 Mayiladuthurai Dist, Tamil Nadu, India

**Keywords:** *Penaeus vannamei*, Broodstock, Specific pathogen free, Quarantine

## Abstract

The introduction of the exotic Pacific whiteleg shrimp, scientifically known as *Penaeus vannamei*, revolutionised India’s shrimp sector and its export landscape since its incorporation into commercial farming in 2009. The cultivation of *P. vannamei* was allowed only with the Specific Pathogen Free (SPF) broodstocks, which were imported from approved overseas SPF broodstock suppliers. The United States, as a pioneer in developing SPF shrimp stocks and a major hub for broodstock companies gradually emerged as the primary supplier of SPF brooders to India. This development significantly contributed to the remarkable surge in shrimp exports from India, which soared from 2000 metric tons in 2008 to 716,004 metric tons in 2024. The consistent brooder importation by India also marked a transformative progression for SPF production companies operating overseas. To ensure biosecurity and provide quarantine services to licensed *P. vannamei* hatcheries that import SPF brooders, the Government of India established a centralized quarantine unit, the Aquatic Quarantine Facility (AQF). This facility serves as the sole entry point for all imported shrimp stocks from various international suppliers. Over the past 16 years, data has been collected through the physical enumeration of the shrimp stocks upon immediate arrival in India at AQF. This data was analyzed using GraphPad Prism version 10.1.0 to understand the import trends and patterns. The data offers valuable insights into the sustainability of the sector and also the dynamic exchange between India and the U.S throughout this period. It also provides a comparative analysis of broodstock imports from non-U.S. countries. This dataset is particularly vital for policy makers and decision-makers, especially in light of the recent announcement regarding reciprocal tariffs by the U.S. Government.

Specifications Table

**Table:** 

Subject	Biology
Specific subject area	Aquaculture
Type of data	Table Graph Raw and analysed data
Data collection	Data was collected at the point of entry of the stocks into the country at AQF through physical enumeration and systematic categorization of the brooders based on the country of origin. The number of male and female brooders imported was counted immediately upon the arrival of each broodstock shipment at AQF and expressed in total quantities. The data spans a period of 16 years and has been systematically organized. Imports from broodstock suppliers in the U.S. have been categorized separately from those originating from countries outside the U.S.
Data source location	Institution: Aquatic Quarantine Facility City: Chennai Region: Tamil Nadu Country: India Latitude and longitude: 12° N 57 24.0942′’ and 80° E 15 56.0664′’.
Data accessibility	Repository name: Mendeley Data identification number: 10.17632/vs679tgx8s.1 Direct URL to data: https://data.mendeley.com/datasets/vs679tgx8s/1
Related research article	None

## 1 Value of the Data

• The dataset provided is the only comprehensive source of information on the importation of *P. vannamei* broodstock into the country. The primary data is collected from the Aquatic Quarantine Facility, which is India’s sole entity responsible for the quarantine of imported shrimp species.
• The dataset offers detailed insights into the extent to which India depends on imported broodstock. Such information is crucial for policymakers to make informed decisions that could shape the future of the shrimp sector in India.
• The data is typically not made available to the general public, academic institutions, or the broader scientific community without direct inquiries to the responsible department. This lack of transparency can hinder collaborative efforts and innovative research.
• Data allows the researchers to analyze the quality and performance of vannamei stocks particularly in terms of their adaptability and success in Indian farm and hatchery environments. Understanding these nuances can significantly influence breeding programs, farming practices, and overall industry standards.
• The historical dataset, being accurate and realistic figures, provides a foundational understanding of vannamei seed production and its related farming practices in India.
• By documenting the origins of these stocks, it enhances traceability, ensuring that stakeholders in the shrimp sector have crucial information that can foster better management practices, improve quality control, and ultimately drive the sector’s growth and sustainability.

## 2 Background

Prior to the introduction of *P. vannamei* culture in India, the country’s shrimp production relied on the native black tiger shrimp, *P. monodon*. This situation continued until a sharp decline in monodon production was witnessed in 1994, due to the outbreak of the White Spot Syndrome Virus (WSSV) and subsequent crop failures. During this period, several Southeast Asian countries including Thailand and Vietnam adopted vannamei culture, due to its advantageous culture attributes such as fast growth, short culture period, high fecundity and tolerance to high salinity and temperature [1]. This transition led to an exponential increase in their shrimp production. Further, the availability of domesticated SPF brooders in the market crucial for mitigating disease risks associated with the transboundary movement of live shrimps [2] urged the India Government to allow the import of SPF *P. vannamei* brooders. After a successful pilot scale culture trial in the country and the establishment of the AQF, commercial culture of *P. vannamei* was initiated. The requirement to exclusively use SPF *P. vannamei* broodstocks for seed production and farming in India [3] made the country overly dependent on international SPF broodstock suppliers. The majority of the stocks utilized for *P. vannamei* culture were imported from U.S based companies. Thus, the U.S which is the largest buyer of Indian shrimps and its value added products emerged as a vital supplier of the live SPF vannamei brooders for India. The expansion of *P. vannamei* culture in India further led the country to heavily rely on U.S for the stocks, although marginal imports from non-U.S. countries were recorded. In addition to live shrimp brooders, the U.S. exports a diverse range of products, including fish and shrimp feed, fresh and chilled shrimp, frozen fish, and artemia cysts to India.

The article presents a dataset on live shrimp imports, which are a critical commodity for the Indian seafood sector and exports. The information highlights the trends in importing shrimp brooders from the U.S. compared to other countries. The data was obtained from India’s sole shrimp quarantine centre, the Aquatic Quarantine Facility (AQF), where all imported shrimp are subjected to rigorous disease screening and quarantine procedures. Established in 2009, the AQF serves the commercial culture of the vannamei sector by providing essential quarantine services to shrimp hatcheries.

Hatcheries licensed to produce *P. vannamei* seeds must import Specific Pathogen Free (SPF) broodstock from designated suppliers empanelled by the Coastal Aquaculture Authority (CAA) with these suppliers located in various regions around the globe. Upon arrival in India, the broodstock is first received at the AQF, where their pathogen-free status is ascertained before handed over to the hatcheries. Over the past 16 years, imports of *P. vannamei* broodstock from diverse sources have been compiled, focusing on two primary categories: imports from the U.S. and imports from non-U.S.. Countries. The import trends for each period, from the two regions, are detailed in this article.

## 3 Data Description

**Fig. 1:**
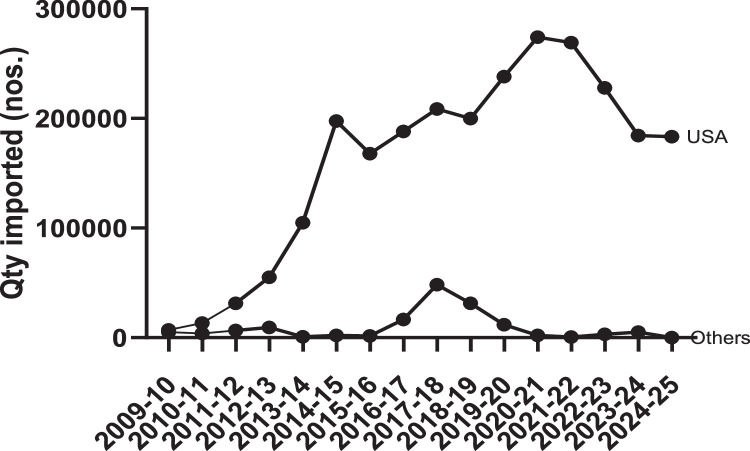
Import of P. vannamei broodstock from the U.S and non-U.S. Countries to India.

A historical dataset detailing the imports of SPF *P. vannamei* brooders from the United States and other countries (non-U.S.) to India over a period of 16 years is presented. The data indicates the quantity of brooders imported by India from various sources. Shrimp stocks supplied from broodstock centers, including Shrimp Improvement Systems (Florida), Shrimp Improvement Systems (Hawaii), American Penaeid (Florida), BenchMark Genetics (Florida), Syaqua Pvt. Ltd. (Florida), Kona Bay Marine Resources (Hawaii), Molokkai Broodstock Company (Florida), Sea Products (later rebranded as Blue Genetics, Texas), and the Oceanic Institute (Hawaii), are categorized under imports from the USA. In contrast, stocks supplied by Shrimp Improvement Systems (Singapore), Blue Genetics (Mexico), Global Gen (Indonesia), Vannamei 101 (Thailand), Syaqua Siam (Thailand), and CP Foods Pvt. Ltd. (Thailand) fall under imports from other countries. Notably, imports from Southeast Asian countries have been banned in India since 2013 [4]; hence, the data from these regions (specifically Thailand-based suppliers) are only included for the years 2009 to 2013. The raw import dataset is available for access at https://data.mendeley.com/datasets/vs679tgx8s/1. Summary statistics of annual imports with Tukey’s Post hoc comparisons are provided in Table 1, Table 2 within the Excel file. The ANOVA summary of the dataset, which analyzes supplies from U.S. and non-U.S. countries, is presented in Table 3. Consolidated data on imports from the two major regions is illustrated in Fig. 1 in the Excel sheet.

**Table 1:** Summary statistsics of *P. vannamei* broodstock supply from the U.S. to India.

Particulars	2009–10	2010–11	2011–12	2012–13	2013–14	2014–15	2015–16	2016–17	2017–18	2018–19	2019–20	2020–21	2021–22	2022–23	2023–24	2024–25
No: of shipments	17	31	70	118	210	312	248	264	283	265	347	146	371	301	297	308
Minimum qty supplied (nos.)	50	81	280	280	120	160	140	200	200	200	200	380	200	159	150	200
Maximum qty supplied (nos.)	1000	1100	1120	840	800	1520	1600	2000	1600	1400	2000	1200	1272	1344	1400	1200
Range	950	1019	840	560	680	1360	1460	1800	1400	1200	1800	820	1072	1185	1250	1000
Mean	387.5^a^	434.9^a^	448^a^	468^a^	499.2^a^	633.2^b^	679.9^b^	720.5^b^	737.5^b^	754.3^b^	686.2^b^	744.2^b^	725.2^b^	690.7^b^	620.6^b^	595^a^
Std. Deviation	233.5	227.6	193.1	176.9	203.1	292.2	313.7	346.2	280.7	274.8	270.6	268.3	279.7	293	236.7	189.1
Std. Error of Mean	56.64	40.88	23.07	16.28	14.02	16.54	19.92	21.31	16.68	16.88	14.52	22.21	14.52	16.89	13.74	10.78
Lower 95 % CI of mean	267.5	351.4	402	435.7	471.6	600.6	640.7	678.5	704.6	721	657.6	700.3	696.6	657.4	593.6	573.8
Upper 95 % CI of mean	507.6	518.4	494	500.2	526.9	665.8	719.1	762.4	770.3	787.5	714.8	788.1	753.7	723.9	647.6	616.2
Total qty imported (nos.)	6588	13,482	31,360	55,220	104,836	197,558	168,618	190,206	208,701	199,880	238,115	108,650	269,034	207,890	184,313	183,266

*Note:* Mean values superscripted with different letters differ significantly (α 0.05 level).

**Table 2:** Summary statistsics of *P. vannamei* broodstock supply from Non-U.S. Countries to India.

Particulars	2009–10	2010–11	2011–12	2012–13	2013–14	2014–15	2015–16	2016–17	2017–18	2018–19	2019–20	2020–21	2021–22	2022–23	2023–24	2024–25
No: of shipments	14	8	13	16	1	3	3	20	3	48	16	4	1	5	6	0
Minimum qty supplied (nos.)	210	217	280	280	800	400	400	400	600	200	200	400	638	400	600	0
Maximum qty supplied (nos.)	600	590	1000	760	800	1200	800	1200	800	1200	1200	600	638	800	1200	0
Range	390	373	720	480	0	800	400	800	200	1000	1000	200	0	400	600	0
Mean	374^c^	356^c^	508^c^	535^c^	800^c^	747^c^	547^c^	726^d^	667^c^	651^d^	738^d^	550^c^	638^c^	608^c^	833^d^	0^c^
Std. Deviation	143	138	229	173	0	411	220	201	115	214	270	100	0	191	294	0
Std. Error of Mean	38.2	48.8	63.5	43.2	0	237	127	45	66.7	30.9	67.6	50	0	85.2	120	0
Lower 95 % CI of mean	291	240	369	443	0	−273	−0.596	632	380	589	593	391		371	524	0
Upper 95 % CI of mean	456	471	646	627	0	1766	1094	820	954	713	882	709		845	1142	0
Total qty imported (nos.)	5229	2847	6600	8560	800	2240	1640	14,520	2000	31,260	11,800	2200	638	3040	5000	0

*Note:* Mean values superscripted with different letters differ significantly (α 0.05 level).

**Table 3:** ANOVA summary of dataset on *P. vannamei* broodstock supply from U.S. and Non-U.S. Countries.

Particulars	U.S.	Non-U.S
F	23.82	4.349
P value	<0.0001	<0.0001
Significant diff. among means (*P* < 0.05)	Yes	Yes
R square	0.09093	0.3088

## 4 Experimental Design, Materials and Methods

Data was directly obtained from the AQF, established by the Marine Products Export Development Authority (MPEDA) under the Government of India. The AQF plays a vital role in mitigating the risks associated with the introduction of *P. vannamei*. As stated in the Indian Government Gazette [3], Chennai International Airport is designated as the sole authorized port of entry for shrimp broodstock in India. Additionally, all *P. vannamei* stocks imported by Indian hatcheries from approved overseas broodstock suppliers [5] must receive certification for their specific pathogen-free (SPF) status from the AQF before being released to the hatcheries. The diseases tested during quarantine include both WOAH-listed [6] and non-listed pathogens. To compile the dataset, broodstocks imported from various countries were systematically enumerated and documented. Mortalities occurring during transit or quarantine were not included in the data; only the total quantity imported over a period of 16 years was considered. The yearly imports were subjected to one-way ANOVA (Table 3) followed by Tukey’s Post hoc test to assess statistical significance. Data analysis was performed using GraphPad Prism version 10.1.0.

## Limitations

Not applicable.

## Ethics Statement

This statement confirms that the authors have read and followed the ethical requirements for publication in Data in Brief and confirms that the current work does not involve human subjects, animal experiments, or any data collected from social media platforms.

## CRediT Author Statement

**M. C. Remany:** Conceptualization, writing and analysis. **Daly Cyriac:** Data collection. **V. Shanmuga Arasu:** Data collection. **Anup Mandal:** Editing and draft finalization.

## Acknowledgements

The authors would like to thank Shri Venkataswamy IAS, Chairman of MPEDA, and Dr S. Kandan, Director of RGCA, for their support and encouragement in preparing this manuscript. They also extend their thanks to the Ministry of Fisheries, the Coastal Aquaculture Authority, the National Fisheries Development Board, and the Central Institute of Brackishwater Aquaculture, for their involvement in reviewing the operations of AQF.

This research did not receive any specific grant from funding agencies in the public, commercial, or not-for-profit sectors.

## Declaration of Competing Interest

The authors declare that they have no known competing financial interests or personal relationships that could have appeared to influence the work reported in this paper.

## Data Availability

Mendeley DataDataset on India’s imports of Penaeus vannamei brooders from the United States (Original data).

## Appendix Supplementary materials

Image, application 1

Image, application 2
